# Brassinosteroid Potentiates Cold-Induced Transcriptome–Metabolome Reprogramming in Tea Plant Leaves: An Integrated Multi-Omics Landscape

**DOI:** 10.3390/ijms27093766

**Published:** 2026-04-23

**Authors:** Wenli Wang, Keyin Shen, Jingbo Yu, Fengshui Yang, Lan Zhang, Shibei Ge, Xin Li

**Affiliations:** 1 Tea Research Institute, Chinese Academy of Agricultural Sciences, Hangzhou 310008, China; 2 State Key Laboratory of Tea Plant Germplasm Innovation and Resource Utilization, Tea Research Institute, Chinese Academy of Agricultural Sciences, Hangzhou 310008, China

**Keywords:** *Camellia sinensis*, 24-epibrassinolide, cold acclimation, multi-omics integration, phenylpropanoid metabolism, hormone, transcription factor

## Abstract

Low temperatures severely restrict tea plant (*Camellia sinensis*) growth and yield stability, yet how brassinosteroid (BR) signaling modulates cold acclimation at a systems level remains insufficiently defined. Here, we integrated transcriptomic and UHPLC–MS metabolomic profiling of tea leaves under Control, 24-epibrassinolide (EBR), Cold, and Cold + EBR treatments to delineate BR-potentiated cold responses. Principal component analyses revealed clear treatment-specific separation and tight clustering of biological replicates at both omics levels. Quantitatively, cold stress induced extensive reprogramming (4075 differentially expressed genes (DEGs) and 298 differentially accumulated metabolites (DAMs)), whereas EBR alone exerted relatively modest effects (231 DEGs and 50 DAMs). Notably, EBR under cold conditions further reshaped cold-responsive networks (371 BR-modulated DEGs and 17 BR-modulated DAMs), consistent with a potentiating role for BR signaling. Functional enrichment analyses highlighted phenylpropanoid metabolism and hormone signal transduction as core responsive modules, with coordinated activation of key gateway genes (*PAL*, *C4H*, and *4CL*) and concurrent engagement of lignin-, flavonoid-, and catechin-associated branches under Cold + EBR. Metabolomic analyses identified flavonoids as the dominant responsive metabolite class (49.31%), particularly anthocyanins and flavonol glycosides. Integrative TF–metabolite–gene correlation networks prioritized WRKY transcription factors (TEA001162, TEA027058) and a UDP-glycosyltransferase gene (TEA025792) as candidate hub genes linking hormone signaling to phenylpropanoid outputs. Collectively, this work provides a systems-level framework of co-regulated transcript–metabolite modules and candidate molecular targets, offering a foundation for functional validation and practical improvement of cold resilience in tea production.

## 1. Introduction

Low temperatures severely restrict the geographical distribution, winter survival, and yield stability of tea plants [*Camellia sinensis* (L.) O. Kuntze], a perennial evergreen crop cultivated across temperate and subtropical regions [1,2]. Episodes of freezing stress compromise photosynthetic efficiency, disrupt cellular redox homeostasis, and cause visible damage to leaves and buds, ultimately reducing harvestable biomass and altering the accumulation of quality-related metabolites [3,4]. To cope with these challenges, tea plants have evolved multilayered acclimation strategies that include membrane remodeling, osmotic adjustment through the accumulation of compatible solutes, and reinforcement of antioxidant capacity [5,6,7]. These adaptive responses are orchestrated by complex phytohormone signaling networks that link environmental perception to growth–defense trade-offs [8,9].

Among phytohormones, brassinosteroids (BRs) have emerged as critical regulatory hubs that modulate abiotic stress tolerance while maintaining developmental programs [10]. BR signaling is initiated at the plasma membrane through the BRI1 receptor and propagated via kinase cascades to transcriptional regulators BZR1/BES1, which function as master switches coordinating growth and stress responses [11]. This coordination is achieved through extensive crosstalk with abscisic acid (ABA), jasmonic acid (JA), and auxin pathways. In particular, BR signaling affects ICE1 stability during cold responses, and BR–JA crosstalk mediated by BES1 contributes to the optimization of the growth–defense balance [12,13]. Crucially, BR signaling does not act as an independent driver but rather functions as a potentiator that amplifies and coordinates endogenous stress-response programs [12,14]. The potentiation of cold tolerance by BR signaling is executed through specific transcription factor (TF) networks that transduce hormonal signals into metabolic adaptations. Group III WRKY transcription factors, such as WRKY46, WRKY54, and WRKY70, participate in BR-mediated signaling to modulate the balance between growth and stress responses [15]. These TFs can function as molecular nodes linking BR signaling with other stress-related pathways, including MAPK cascades, thereby regulating downstream target genes involved in cellular protection [16]. Additionally, R2R3-MYB and bHLH transcription factors often assemble with WD40 proteins into conserved MBW (MYB–bHLH–WD40) complexes that regulate structural genes in flavonoid biosynthesis [17,18]. BR signaling can further modulate this regulatory module through BES1/BZR1-dependent regulation of flavonoid-related MYB factors and, in some contexts, through cooperation with anthocyanin regulatory factors [19,20].

Together, these TF hubs provide a mechanistic basis for understanding how BR signaling is translated into downstream metabolic reprogramming under stress conditions. Among the diverse metabolic pathways orchestrated by BR-responsive TFs, phenylpropanoid metabolism represents a major downstream metabolic output of cold protection strategies. This pathway connects primary metabolism to the production of specialized defensive compounds, including lignin for cell wall reinforcement and flavonoids (catechins, flavonols, anthocyanins) that function as potent reactive oxygen species (ROS) scavengers and membrane stabilizers [21,22]. Cold-resistant tea cultivars consistently accumulate higher levels of phenylpropanoid-derived metabolites, particularly catechins and flavonol glycosides, which contribute to both stress tolerance and tea quality attributes [23,24,25]. The biosynthesis of these compounds is initiated by gateway enzymes PAL, C4H, and 4CL, and branches into lignin, flavonoid, and catechin pathways that collectively determine cellular redox status under low temperature [26,27].

Despite the well-documented association between phenylpropanoid accumulation and cold tolerance, the systems-level mechanisms by which BR signaling regulates this metabolic hub through TF networks in tea plants remain poorly understood. In particular, it is still unclear how exogenous EBR enhances cold-responsive transcriptional programs to redirect carbon flux toward protective phenylpropanoid metabolism, and which TF hubs mediate this hormone-to-metabolite transition. We therefore hypothesized that exogenous EBR promotes cold acclimation in tea plants by strengthening BR-associated transcriptional regulation and reprogramming phenylpropanoid metabolism through specific TF networks. To test this hypothesis, we performed integrated transcriptomic and UHPLC–MS metabolomic analyses across four treatments (Control, EBR, Cold, and Cold + EBR) to delineate the multi-omics landscape of BR-potentiated cold acclimation. Specifically, this study aimed to (i) define the global transcriptional signatures associated with EBR application under cold stress, (ii) identify the hierarchical regulation of phenylpropanoid metabolism by BR-responsive transcription factors, and (iii) construct a mechanistic framework linking BR signaling, TF-mediated transcriptional control, and metabolic remodeling. By combining pathway enrichment, gene–metabolite correlation networks, this work seeks to provide molecular targets for breeding strategies aimed at enhancing cold resilience in tea production.

## 2. Results

### 2.1. Transcriptomic and Metabolomic Analyses of Tea Leaves Treated with EBR Under Low Temperature Stress

**Figure 1:**
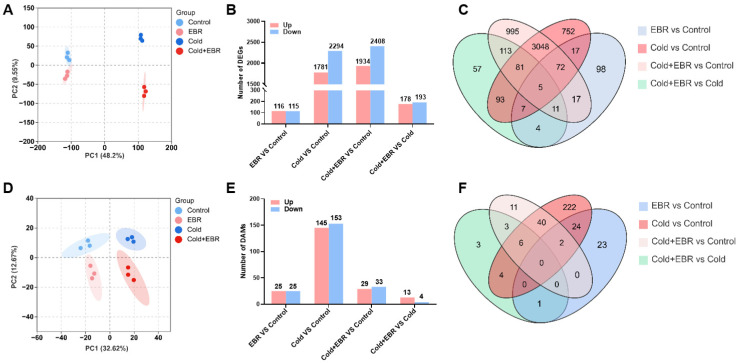
Transcriptomic and metabolomic profiling of tea plants under BR and cold treatments. (**A**) Principal component analysis (PCA) of transcriptomic samples. Different colors indicate different treatment groups, and each point represents one biological replicate. (**B**) Numbers of up-regulated and down-regulated differentially expressed genes (DEGs) identified in different pairwise comparisons. (**C**) Venn diagram showing the overlap of DEGs among the four comparison groups. (**D**) Principal component analysis (PCA) of metabolomic samples. Different colors indicate different treatment groups, and each point represents one biological replicate. (**E**) Numbers of increased and decreased differentially accumulated metabolites (DAMs) identified in different pairwise comparisons. (**F**) Venn diagram showing the overlap of DAMs among the four comparison groups.

To elucidate the role of EBR in the response of tea plants to low temperature, integrated transcriptomic and metabolomic analyses were performed under four treatments: Control, EBR, Cold, and Cold + EBR. Transcriptome sequencing generated high-quality data for all samples, with Q30 values above 96% and GC contents of approximately 45%, supporting the reliability of the subsequent analyses (Table S1). Principal component analysis (PCA) of the transcriptomic data showed tight clustering of biological replicates and clear separation among treatments, indicating high reproducibility and distinct transcriptional responses (Figure 1A). PC1 and PC2 explained 48.2% and 9.55% of the total variance, respectively. Differential expression analysis identified 231, 4075, 4342, and 371 differentially expressed genes (DEGs) in EBR vs. Control, Cold vs. Control, Cold + EBR vs. Control, and Cold + EBR vs. Cold, respectively (Figure 1B). Venn analysis further revealed that 27 DEGs were shared between the EBR-responsive gene sets under normal- and low-temperature conditions, whereas 186 DEGs overlapped between the cold-responsive and EBR-modulated gene sets (Figure 1C). These results suggest that cold stress triggered extensive transcriptional reprogramming, while EBR treatment under cold conditions further altered the expression of a subset of cold-responsive genes.

Metabolomic PCA revealed a similar separation pattern among the four treatments, indicating that metabolite profiles were also markedly affected by EBR and low temperature (Figure 1D). In total, 50, 298, 62, and 17 differentially accumulated metabolites (DAMs) were identified in EBR vs. Control, Cold vs. Control, Cold + EBR vs. Control, and Cold + EBR vs. Cold, respectively (Figure 1E). Among these, 1 DAM was commonly regulated by EBR under both normal- and low-temperature conditions, and 24 DAMs overlapped between the cold-responsive and EBR-modulated metabolite sets (Figure 1F). Collectively, these data indicate that low temperature accounted for most of the transcriptomic and metabolomic alterations observed in tea plants, whereas exogenous EBR further reshaped a specific subset of cold-associated molecular responses.

### 2.2. KEGG Enrichment Analysis of Tea Plant Metabolites Under Different Treatments

**Figure 2:**
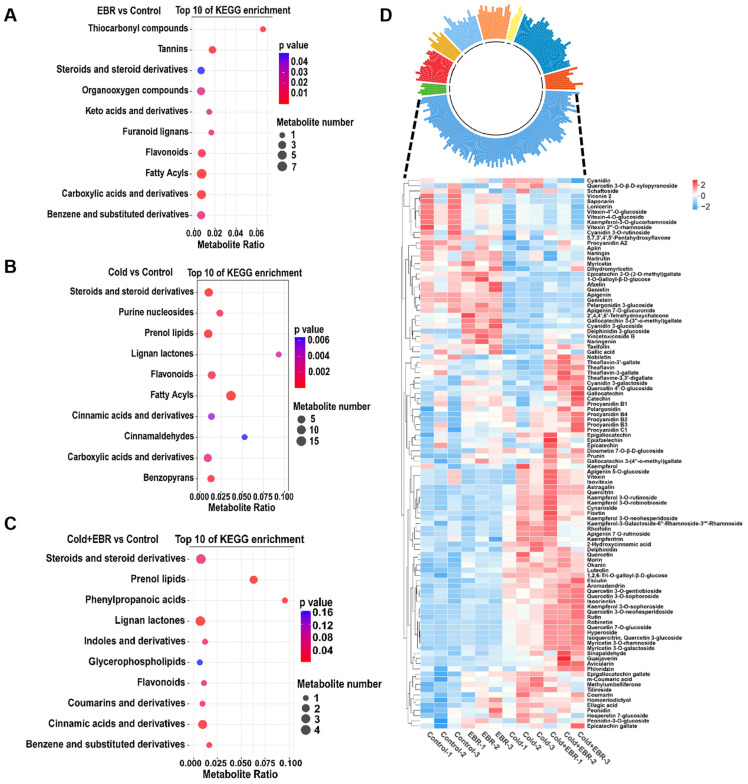
Metabolomic profiling and KEGG enrichment analysis under different treatments. (**A**–**C**) KEGG enrichment analyses of differentially accumulated metabolites (DAMs) in the comparisons of EBR vs. Control, Cold vs. Control, and Cold + EBR vs. Control, respectively. (**D**) Circular plot showing the composition of annotated metabolites in tea leaves (**top**) and heatmap displaying the relative abundance of flavonoid-related metabolites across different treatment groups (**bottom**).

To characterize metabolic processes responsive to cold stress and EBR treatment, metabolite classification and KEGG enrichment analyses were performed. Metabolite profiling showed that stress-responsive changes involved multiple metabolite classes. KEGG enrichment analysis of DAMs revealed that EBR treatment significantly enriched thiocarbonyl compounds, tannins, steroids and steroid derivatives, and related metabolite classes, whereas cold stress predominantly affected steroids and steroid derivatives, purine nucleosides, prenol lipids, and pathways associated with secondary metabolism (Figure 2A,B). Under low-temperature conditions, EBR further enhanced the enrichment of pathways associated with stress-adaptive metabolism, particularly those related to steroids and steroid derivatives as well as prenol lipids (Figure 2C). These results suggest that EBR treatment under cold conditions reinforces metabolic remodeling across multiple stress-responsive processes rather than acting through a single metabolite class.

Among the annotated metabolites, flavonoids (49.31%), amino acids (15.21%), organoheterocyclic compounds (7.83%), nucleosides and nucleotides (7.37%), and organooxygen compounds (7.37%) were the most abundant classes. Among these, flavonoid-related metabolites were the only group consistently represented among the top enriched KEGG categories in EBR vs. Control, Cold vs. Control, and Cold + EBR vs. Control (Figure 2A–C). Hierarchical clustering of flavonoid-related metabolites showed that anthocyanin-type flavonoids were induced by EBR under both normal- and low-temperature conditions, whereas flavonol glycosides were strongly induced by low temperature and were further enhanced by EBR application (Figure 2D).

### 2.3. GO and KEGG Pathway Enrichment Analysis of DEGs with EBR Under Low Temperature Stress

**Figure 3:**
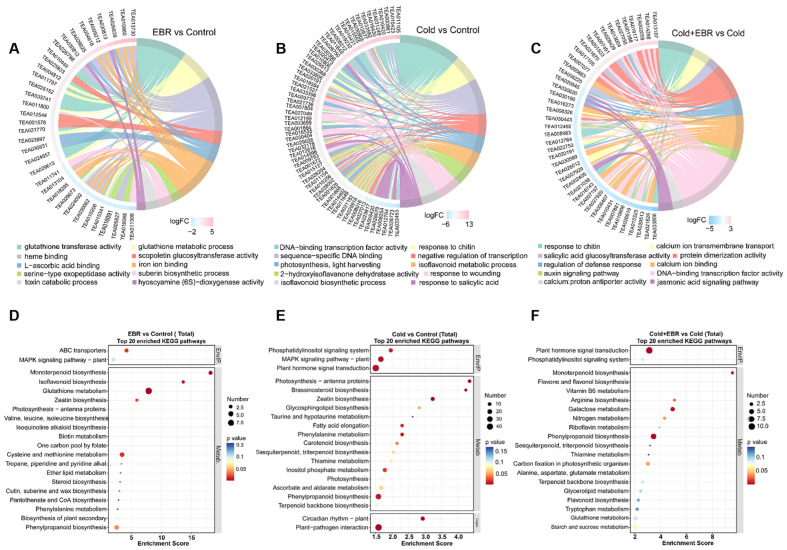
GO and KEGG enrichment analyses of differentially expressed genes (DEGs) under different treatments. (**A**–**C**) Chord diagrams showing the top 10 enriched GO terms in the comparisons of EBR vs. Control, Cold vs. Control, and Cold + EBR vs. Cold, respectively. The outer sectors represent enriched GO terms and their associated genes, the connecting ribbons indicate gene–term associations, and the outer color track represents the logFC values of the corresponding genes. (**D**–**F**) Bubble plots showing the top 20 enriched KEGG pathways in the comparisons of EBR vs. Control, Cold vs. Control, and Cold + EBR vs. Cold, respectively. The *x*-axis indicates the enrichment score, bubble size represents the number of DEGs enriched in each pathway, and bubble color indicates the *p* value.

GO and KEGG enrichment analyses revealed distinct enrichment patterns across the three comparisons (Figure 3). In EBR vs. Control, DEGs were predominantly enriched in GO terms related to detoxification and secondary metabolism, including glutathione transferase activity (such as genes TEA007359 and TEA015130), toxin catabolic process (such as gene TEA000036), and L-ascorbic acid binding (such as gene TEA002994), while KEGG analysis highlighted glutathione metabolism and isoflavonoid biosynthesis (Figure 3A,D). In Cold vs. Control, the enriched terms were mainly associated with transcriptional regulation and photosynthesis-related processes, including DNA-binding transcription factor activity (including genes TEA022042, TEA013019, and TEA010501) and photosynthesis/light harvesting (such as TEA004892 and TEA018642), with KEGG pathways enriched in plant hormone signal transduction, phenylpropanoid biosynthesis, and brassinosteroid biosynthesis (Figure 3B,E). In Cold + EBR vs. Cold, enrichment further highlighted hormone signaling-related processes, including auxin-activated signaling pathway, jasmonic acid-mediated signaling pathway, and salicylic acid glucosyltransferase activity, together with transcription factor activity; the corresponding KEGG pathways included plant hormone signal transduction, phosphatidylinositol signaling, and flavone and flavonol biosynthesis (Figure 3C,F).

Collectively, these results indicate that EBR treatment was mainly associated with redox- and secondary metabolism-related responses, cold stress primarily affected transcriptional regulation and hormone-related pathways, and EBR application under cold conditions further enhanced hormone signaling and transcription-associated processes.

### 2.4. Identification of DEGs Involved in Phenylpropanoid Biosynthesis

**Figure 4:**
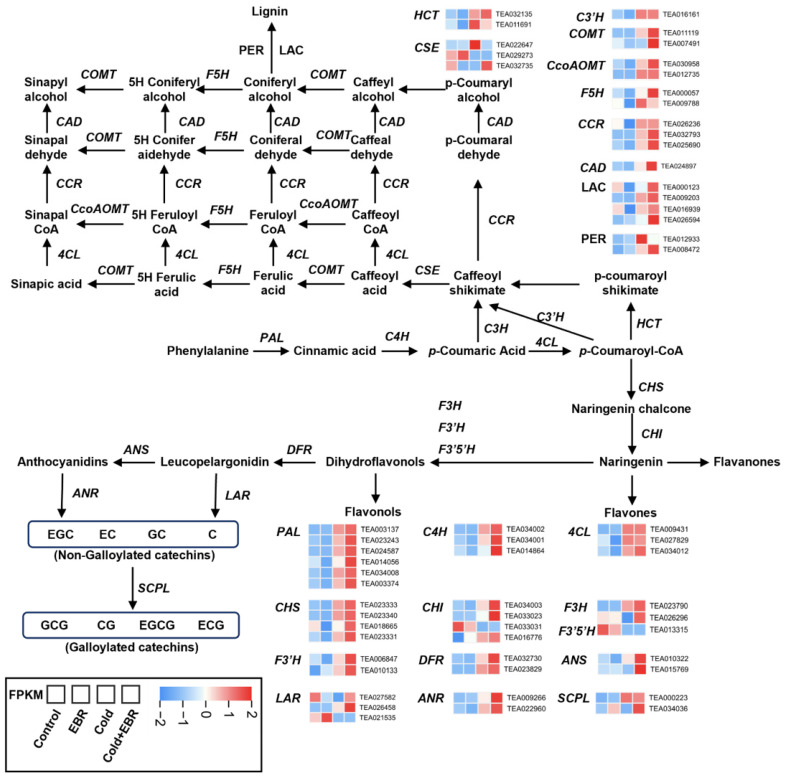
Expression profiles of phenylpropanoid pathway-related genes under different treatments. Embedded heatmaps display the expression patterns of genes encoding key enzymes involved in these pathways. Heatmap values were calculated from FPKM data and further subjected to row-wise normalization. Blue and red represent relatively low and high transcript abundanace, respectively.

To characterize phenylpropanoid-centered transcriptional reprogramming, we mapped DEGs onto the phenylpropanoid biosynthesis pathway (Figure 4). At the upstream steps of the pathway, genes encoding *PAL* (TEA003137, TEA034008), *C4H* (TEA034002, TEA034001), and *4CL* (TEA009431, TEA034012) were broadly upregulated under cold stress, with expression further increased in Cold + EBR. Genes involved in the formation of caffeoyl/feruloyl intermediates, including *HCT* (TEA032135), *C3′H* (TEA016161), and *CSE* (TEA022647), exhibited similar cold-induced expression patterns that were further reinforced by EBR application.

In downstream branches, lignin-associated genes showed marked responsiveness to cold stress, particularly *CCR* (TEA032793, TEA026236), *CAD* (TEA024897), and *PER* (TEA012933), and their expression levels were further elevated in Cold + EBR. In parallel, the flavonoid branch displayed coordinated activation: *CHS* (TEA023333, TEA023340), *CHI* (TEA034003, TEA033023), *F3H* (TEA023790), and *F3′H* (TEA006847) were strongly induced by cold and further enhanced by EBR, as were the downstream genes *DFR* (TEA032730) and *ANS* (TEA010322). Meanwhile, catechin biosynthesis genes, including *ANR* (TEA009266), *LAR* (TEA027582, TEA021535), and *SCPL* (TEA000223, TEA034036), also showed pronounced upregulation under the combined treatment. These results indicate that cold stress induced broad activation of phenylpropanoid metabolism, and that this response was further intensified by EBR, encompassing upstream pathway genes as well as lignin-, flavonoid-, and catechin-associated branches.

### 2.5. Expression Profiles of Phytohormone Signal Transduction Related DEGs

**Figure 5:**
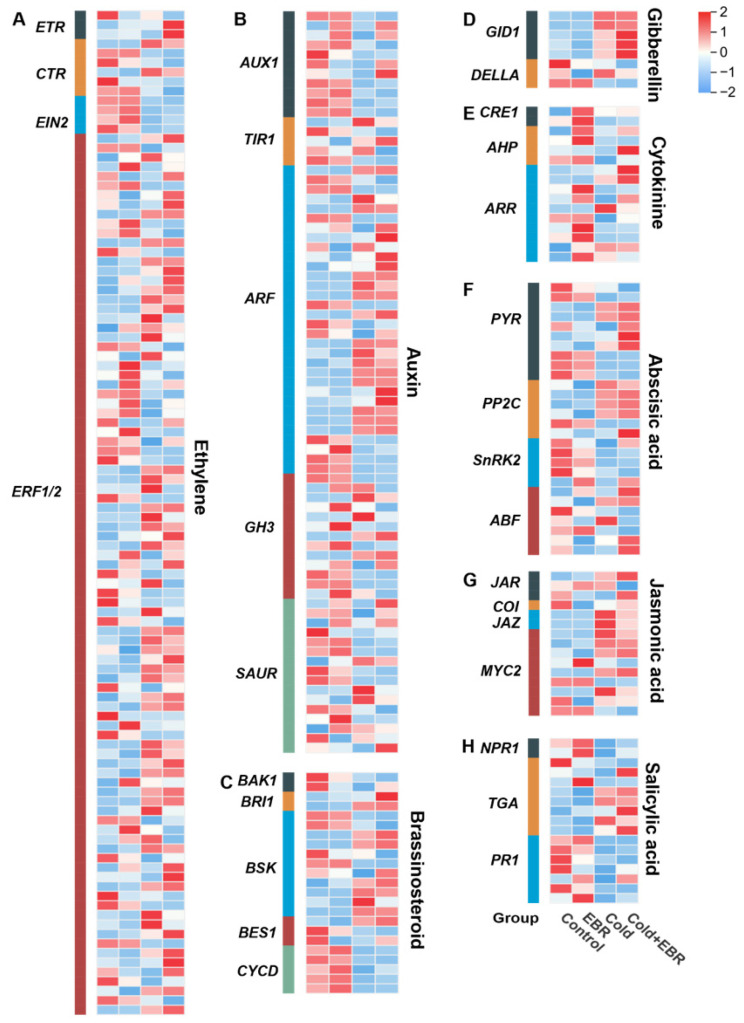
Heatmap of differentially expressed genes (DEGs) involved in phytohormone signal transduction pathways. (**A**–**H**) Expression profiles of DEGs related to ethylene, auxin, brassinosteroid, gibberellin, cytokinin, abscisic acid, jasmonic acid, and salicylic acid signaling pathways, respectively. Columns represent the Control, EBR, Cold, and Cold + EBR treatments. Heatmap values were generated from FPKM data and further normalized in a row-wise manner. Red and blue indicate relatively high and low expression levels, respectively.

To further characterize the transcriptional reprogramming of phytohormone signaling under EBR and low-temperature treatments, we analyzed the expression patterns of DEGs involved in eight phytohormone signaling pathways (Figure 5) in conjunction with the enrichment results shown in Figure 3. Overall, hormone-associated DEGs exhibited clear pathway-specific and treatment-dependent expression patterns, and the combined Cold + EBR treatment generated a transcriptional profile distinct from either EBR or cold treatment alone.

EBR treatment alone prominently activated growth-associated hormonal modules. Auxin signaling genes, including *AUX1*, *TIR1*, *ARF*, *GH3*, and *SAUR*, showed elevated transcript abundance, together with brassinosteroid pathway genes (*BRI1*, *BAK1*, *BSK*, *BES1*, *CYCD*) and cytokinin-responsive genes (*CRE1*, *AHP*, *ARR*), consistent with the enrichment of auxin-activated signaling observed in Cold + EBR vs. Cold (Figure 3C).

Cold stress triggered broad reprogramming of stress-responsive hormone networks. Abscisic acid signaling genes (*PYR*, *PP2C*, *SnRK2*, *ABF*) and ethylene pathway components (*ETR*, *CTR*, *EIN2*, *ERF1/2*) displayed marked cold-induced expression, consistent with the KEGG enrichment of plant hormone signal transduction in Cold vs. Control (Figure 3E). In addition, gibberellin-related genes (*GID1*, *DELLA*) also showed altered expression patterns under low temperature.

Under the combined Cold + EBR treatment, defense-related hormone signaling was further intensified. JA pathway genes (*JAR*, *COI*, *JAZ*, *MYC2*) and salicylic acid (SA) signaling components (*NPR1*, *TGA*, *PR1*) exhibited higher expression levels than under cold treatment alone, consistent with the enrichment of jasmonic acid-mediated signaling and salicylic acid-related glucosyltransferase activity in Cold + EBR vs. Cold (Figure 3C,F). Taken together, these findings indicate that EBR and cold stress cooperatively reprogram the hormone regulatory network. Cold stress primarily triggers stress-associated hormonal reprogramming, whereas exogenous EBR further reinforces JA- and SA-related signaling while partially maintaining growth-related hormone pathways, thereby helping to balance stress adaptation with growth regulation.

### 2.6. Integrated Correlation Analysis of Key Metabolites and Genes with Validation of Genes

**Figure 6:**
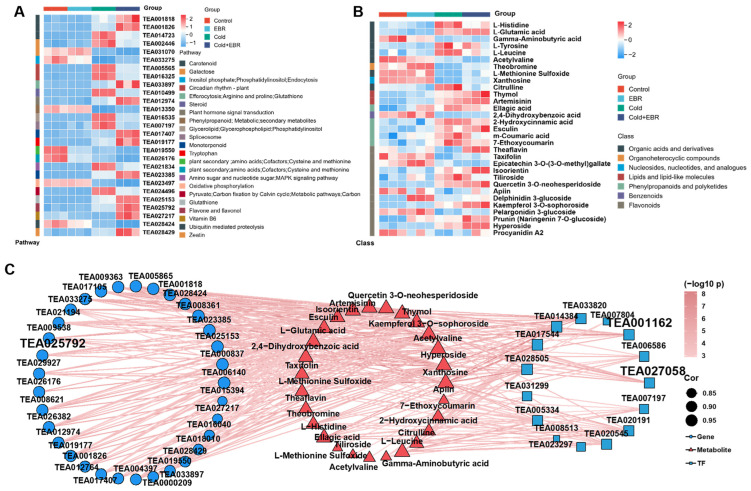
Heatmaps and correlation network analysis of differentially expressed genes (DEGs), differentially accumulated metabolites (DAMs), and transcription factors (TFs) under BR and cold treatments. (**A**) Heatmap showing the expression profiles of DEGs across the Control, EBR, Cold, and Cold + EBR treatments. (**B**) Heatmap showing the accumulation patterns of DAMs across the Control, EBR, Cold, and Cold + EBR treatments. (**C**) Correlation network integrating DEGs, TFs, and DAMs. Circles, squares, and triangles represent genes, TFs, and metabolites, respectively. The color gradient indicates significance as −log_10_(*p*), and the correlation strength is shown according to the legend. Gene heatmap values were generated from FPKM data and further normalized in a row-wise manner.

To investigate coordinated transcriptomic–metabolomic responses, we examined the expression patterns of selected DEGs and the accumulation profiles of DAMs. Genes associated with secondary metabolism, signal transduction, and phytohormone signaling exhibited pronounced transcriptional reprogramming under Cold and Cold + EBR treatments, whereas EBR alone exerted comparatively moderate effects (Figure 6A). Consistently, metabolite profiling revealed that multiple classes, including amino acids (L-glutamic acid, gamma-aminobutyric acid), organic acids, and phenylpropanoid derivatives (quercetin 3-*O*-neohesperidoside, kaempferol 3-*O*-sophoroside), were responsive to low temperature, with Cold + EBR showing particularly prominent accumulation of flavonol-related compounds (Figure 6B).

Detailed inspection of hub-associated genes revealed distinct expression trajectories across treatments. WRKY transcription factor genes TEA001162 and TEA027058 (Figure S1), together with the UDP-glycosyltransferase gene TEA025792, showed marked upregulation under Cold + EBR relative to cold treatment alone. Phenylpropanoid pathway genes, including *PAL* (TEA003137), *4CL* (TEA034012), *CHS* (TEA023333), and *CHI* (TEA034003), were induced by cold stress and further upregulated following EBR application. These expression patterns were further validated by qRT–PCR (Figure S2), and the qRT–PCR results were consistent with the RNA-seq data for all 12 selected candidate genes, including TEA024587, TEA019177, TEA017407, TEA028429, TEA001818.

Gene–metabolite correlation network analysis (Figure 6C) prioritized candidate hub genes showing strong associations (|PCC| ≥ 0.9) with phenylpropanoid-related and stress-responsive metabolite modules. Notably, TEA001162 and TEA027058 (WRKY family), as well as TEA025792 (UGT), showed strong positive correlations with flavonol derivatives, benzoic acid derivatives (2,4-dihydroxybenzoic acid), and specific amino acid metabolites (L-methionine sulfoxide). qRT–PCR validation further confirmed the consistency between RNA-seq and qRT–PCR data for these hub genes and phenylpropanoid pathway genes, supporting the coordinated regulation of phenylpropanoid-centered modules under EBR-potentiated cold stress (Figure S2).

## 3. Discussion

### 3.1. EBR Acts as a Potentiator of Cold-Induced Transcriptional and Metabolic Reprogramming

Cold stress severely restricts tea plant growth and yield by impairing photosynthetic efficiency, disrupting cellular redox homeostasis, and causing extensive membrane damage [28]. To cope with these challenges, tea plants activate multilayered acclimation strategies involving both transcriptional reprogramming and metabolic remodeling [5,6]. Although BRs are known to enhance abiotic stress tolerance, the extent to which BR signaling modulates cold acclimation at the systems level—particularly by amplifying endogenous defense programs rather than acting independently—remains insufficiently understood [29]. In the present study, cold treatment induced extensive reprogramming in tea leaves, as reflected by 4075 DEGs and 298 DAMs relative to the control (Figure 1B,E), with clear separation among treatments in the PCA analyses (Figure 1A,D).

Previous studies have shown that steroidal plant hormones, BRs, participate not only in the regulation of plant growth and development but also in enhancing tolerance to both biotic and abiotic stresses [29,30]. In this study, exogenous EBR application under control temperature induced only relatively limited transcriptional and metabolic changes, with 231 DEGs and 50 DAMs identified. By contrast, under cold conditions, EBR treatment was associated with an increased number of cold-responsive DEGs and DAMs, including 4342 total DEGs and 62 total DAMs in Cold + EBR vs. Control, of which 371 DEGs and 17 DAMs were specifically altered in Cold + EBR vs. Cold (Figure 1B,C,E,F). These patterns suggest that BR signaling primarily acts to potentiate and coordinate endogenous cold-response programs rather than functioning as an independent regulator that overrides basal cold signaling. Similar synergistic effects of BR signaling have been reported in *Arabidopsis* and tomato, where BR signaling exerts relatively mild effects on transcriptional regulation under optimal growth conditions but plays a critical role in maintaining cellular homeostasis and enhancing stress tolerance under adverse conditions [31,32]. Mechanistically, this potentiation has been linked to the core BR transcription factor BZR1, which promotes freezing tolerance through both CBF-dependent and CBF-independent pathways in *Arabidopsis*, as well as to BR signaling components that regulate the stability of ICE1, a central regulator of cold responses [12,14].

At the pathway level, this potentiating effect was reflected in the coordinated enrichment of stress-adaptive modules. KEGG analysis showed that cold stress predominantly activated plant hormone signal transduction and phenylpropanoid biosynthesis (Figure 3E), whereas the combined Cold + EBR treatment further intensified auxin-activated signaling, jasmonic acid-mediated pathways, and phosphatidylinositol signaling (Figure 3C,F), consistent with enhanced hormone crosstalk under BR modulation. The concurrent activation of steroid- and phenylpropanoid-related metabolism under Cold + EBR (Figure 2C) further supports the view that EBR enhances metabolic remodeling toward protective pathways, including the flavonoid and lignin branches (Figure 4), thereby contributing to cellular protection against cold-induced oxidative damage.

### 3.2. Pathway-Wide Activation of Phenylpropanoid Metabolism Under BR-Potentiated Cold Stress

Among the diverse transcriptional and metabolic changes potentiated by EBR under cold stress, phenylpropanoid metabolism emerged as one of the most prominently enriched pathways, suggesting that the enhancement of cold tolerance is accompanied by a shift in metabolic output toward specialized metabolite classes. KEGG enrichment analysis revealed that phenylpropanoid biosynthesis ranked among the top pathways in both Cold vs. Control and Cold + EBR vs. Control comparisons (Figure 3E), with concurrent enrichment of flavone and flavonol biosynthesis specifically in Cold + EBR vs. Cold (Figure 3F). Metabolomic profiling further supported these findings, showing that flavonoids constituted the predominant class of cold-responsive metabolites, accounting for 49.31% of the annotated differential metabolites, and that anthocyanin-type flavonoids and flavonol glycosides accumulated markedly under Cold + EBR conditions (Figure 2D).

Pathway projection analysis (Figure 4) further showed that this metabolic remodeling involved coordinated transcriptional activation across major branches of the phenylpropanoid pathway rather than selective induction of only terminal branches. At the upstream steps of the pathway, genes encoding *PAL* (TEA003137), *C4H* (TEA034002), and *4CL* (TEA009431) exhibited robust cold-induced expression that was further enhanced by EBR. This upstream activation was accompanied by increased expression of lignin-associated genes, including *CCR* (TEA032793), *CAD* (TEA024897), and *PER* (TEA012933), consistent with enhanced cell wall-related responses. At the same time, the flavonoid branch displayed coordinated upregulation of *CHS* (TEA023333), *CHI* (TEA034003), *F3H* (TEA023790), and *DFR* (TEA032730), in line with enhanced antioxidant-related capacity. Notably, catechin-associated genes, including *ANR* (TEA009266), *LAR* (TEA027582), and *SCPL* (TEA000223), showed synergistic induction under Cold + EBR, indicating the activation of multiple phenylpropanoid-derived protective outputs encompassing both structural (lignin) and biochemical (flavonoid/catechin) defenses.

This pathway-wide activation is consistent with mechanisms reported in diverse plant species, in which cold stress triggers CBF-dependent upregulation of phenylpropanoid biosynthesis genes [21,25]. Recent evidence further indicates that ICE1-related transcription factors enhance freezing tolerance and are associated with the activation of phenylpropanoid biosynthesis and starch–sucrose metabolism [33]. Moreover, chilling stress is frequently accompanied by the induction of key phenylpropanoid- and flavonoid-pathway genes, including *PAL*, *CHS*, *4CL*, and *CCR*, together with increased accumulation of protective phenylpropanoid metabolites [22,34]. The BR-mediated modulation of this pathway observed here is also consistent with findings in sorghum, where *SbBRI1* receptor mutants display altered phenylpropanoid metabolism, including reduced lignin precursors and changes in flavonoid composition [35]. Unlike studies reporting preferential accumulation of specific flavonoid subclasses under cold stress [21], our results indicate that EBR promoted broad activation of phenylpropanoid metabolism, concurrently enhancing lignin, flavonoid, and catechin branches (Figure 4 and Figure 6B). This broad pathway engagement, together with the strong gene–metabolite correlations observed between WRKY transcription factors TEA001162 and TEA027058 and flavonol derivatives (Figure 6C), suggests that BR signaling potentiates a multifaceted metabolic strategy integrating cell wall reinforcement with redox buffering during cold acclimation.

### 3.3. Hormonal Crosstalk Integrates BR Signaling with Classical Cold-Response Pathways

Given that phenylpropanoid metabolism is subject to complex hormonal regulation, we next investigated how EBR modulates phytohormone signaling networks to orchestrate the metabolic reprogramming observed under low-temperature conditions. Transcriptomic analysis revealed that hormone signal transduction pathways were prominently enriched under cold stress, with distinct modulation patterns observed in Cold + EBR compared with cold treatment alone (Figure 3E,F). Specifically, GO enrichment in Cold + EBR vs. Cold highlighted auxin-activated signaling, jasmonic acid-mediated signaling, and salicylic acid-related glucosyltransferase activity (Figure 3C), suggesting that EBR potentiates cold acclimation through integrated remodeling of growth- and defense-related hormone circuits. This is consistent with accumulating evidence that BR signaling acts as a central integrator, modulating the balance between growth-promoting pathways, such as auxin and cytokinin, and stress-responsive pathways, including ABA, JA, and SA, under low-temperature conditions [14,30].

Mechanistically, crosstalk between BR and JA signaling appears to play an important role in cold stress adaptation. In the present study, JA pathway genes including *JAR*, *COI*, *JAZ*, and the key transcription factor MYC2 exhibited enhanced expression under Cold + EBR relative to cold treatment alone (Figure 5G). The bHLH transcription factor MYC2 functions as a master regulator of JA signaling and is released from JAZ-mediated repression following JA-Ile-dependent formation of the COI1–JAZ co-receptor complex [36,37]. Recent studies have shown that BR signaling components physically interact with JA-related regulators to modulate stress responses. For example, in apple (*Malus × domestica*), the BR-responsive transcription factor BIM1 forms a complex with CBF2 to enhance COR gene expression, whereas JAZ proteins interact with BIM1 and interfere with BIM1–CBF2 complex formation, thereby establishing a regulatory switch that integrates BR and JA signaling during cold tolerance [38]. Similarly, in pear (*Pyrus pyrifolia*), PpyBZR2 directly interacts with PpyMYC2 and cooperatively activates gibberellin biosynthetic genes, thereby promoting dormancy release [39]. Together, these findings suggest that the coordinated induction of *MYC2* and BR signaling genes such as *BRI1* and *BZR1* observed in our study (Figure 5C,G) may reflect a conserved mechanism by which BR–JA convergence enhances transcriptional outputs associated with cold tolerance.

Concurrently, BR signaling also intersects with ABA-dependent stress responses through the regulation of key transcription factors. Under cold stress, ABA signaling components, including the receptors *PYR*, phosphatases *PP2C*, kinases *SnRK2*, and downstream bZIP transcription factors *ABF*, showed marked induction (Figure 5F), consistent with the established ABA–PYR/PYL–PP2C–SnRK2–ABF signaling cascade that activates stress-responsive genes, including cold-responsive transcriptional programs [40,41,42]. The BR signaling kinase BIN2, a negative regulator of BZR1/BES1, has been shown to phosphorylate and destabilize ICE1, a central regulator of *CBF* expression, whereas BR-induced inactivation of BIN2 during early cold stress facilitates BZR1 accumulation and CBF activation [12]. This temporal regulation suggests that BR signaling may fine-tune ABA-mediated stress responses by modulating the stability and activity of shared transcriptional nodes. Furthermore, WRKY family transcription factors identified here as candidate hub genes in the gene–metabolite correlation network (TEA001162, TEA027058) are known to participate in hormone crosstalk involving SA and ABA. Accumulating evidence further indicates that certain WRKYs contribute to cold-stress responses through ABA-related regulatory pathways, potentially linking hormone crosstalk to the phenylpropanoid metabolic remodeling observed in Cold + EBR (Figure 4 and Figure 6C) [43,44,45]. Collectively, these findings support the view that EBR enhances cold tolerance by orchestrating transcription factor networks, particularly MYC2-mediated JA signaling, ABF-mediated ABA responses, and WRKY-associated defense outputs, thereby coordinating growth restraint, ROS homeostasis, and secondary metabolite accumulation.

### 3.4. Transcription Factors Mediate BR Signaling in Cold Tolerance Regulation

TFs serve as important regulatory nodes that potentially transduce upstream hormonal signals into downstream metabolic outputs during cold acclimation. In the present study, integrated gene–metabolite correlation analysis identified multiple candidate hub genes, including WRKY transcription factors (TEA001162, TEA027058) and additional members of the MYB and bHLH families, together with a UDP-glycosyltransferase gene (TEA025792). These hub genes showed strong associations with phenylpropanoid-related metabolites, particularly flavonol glycosides, benzoic acid-related compounds, and amino acid-related metabolites, suggesting that they may participate in linking BR-associated hormone signaling with secondary metabolic remodeling. However, it should be noted that these relationships are mainly inferred from co-expression and correlation analyses and thus do not constitute direct evidence of regulatory or causal interactions. Among these candidates, WRKY transcription factors were of particular interest because of their established roles in stress signaling and developmental regulation. In the present study, TEA001162 and TEA027058 were further induced by EBR under cold conditions, and their expression patterns were consistent with the qRT–PCR results. WRKY transcription factors are widely recognized as regulators of abiotic stress tolerance and as important nodes of signaling crosstalk. For example, in *Arabidopsis*, group III WRKYs participate in BR-mediated signaling to coordinate growth and drought responses, whereas in rice, WRKY53 mediates crosstalk between BR signaling and the MAPK pathway to regulate stress-related developmental traits [15,46]. In this context, the close associations of TEA001162 and TEA027058 with flavonol glycosides and phenylpropanoid intermediates support their potential roles as upstream regulators of phenylpropanoid metabolism. Nevertheless, whether these tea WRKYs directly bind the promoters of structural genes through W-box elements, or instead act indirectly through interaction with other TFs, remains unresolved in the current study.

Concurrently, UDP-glycosyltransferases (UGTs) and R2R3-MYB/bHLH TFs may also contribute to the metabolic specialization observed under Cold + EBR treatment. The UGT gene TEA025792 exhibited significant co-expression with flavonol derivatives, which is consistent with the established roles of UGTs in stabilizing flavonoids through glycosylation and thereby enhancing ROS-scavenging capacity [47,48]. In tea plants, *CsUGT78A14* and *CsUGT78A15* are specifically induced by cold stress and catalyze the formation of flavonol 3-*O*-glucosides/galactosides that contribute to cold tolerance [48,49]. In addition, MYB and bHLH TFs are likely to participate in MBW (MYB–bHLH–WD40) complexes that regulate phenylpropanoid flux. In blueberry and apple, cold stress induces MYB14, MYB4, and bHLH TFs that coordinate with WRKYs to activate phenylpropanoid biosynthesis genes [50,51]. Based on these observations, the concurrent induction of these TFs and genes under Cold + EBR is consistent with a layered regulatory architecture in which WRKYs may integrate hormone-related signals, MYB/bHLH complexes may regulate structural genes, and UGTs may modify downstream metabolites to optimize cellular protection. However, these inferred roles should still be considered putative until supported by direct functional evidence.

Taken together, our data support a working model in which EBR is associated with enhanced cold tolerance through coordinated transcriptional and metabolic reprogramming involving candidate WRKY-, MYB/bHLH-, and UGT-related modules. Importantly, this model is based on multi-omics integration and correlation analysis, and thus remains provisional at the mechanistic level. Future studies should incorporate functional validation approaches, such as gene knockdown, knockout, or overexpression analyses, combined with promoter-binding and transactivation assays, to verify the causal roles of these candidate hub genes in BR-mediated cold responses. Such experiments will be essential for determining whether the identified WRKY TFs and UGT truly function as central regulators of phenylpropanoid remodeling during cold acclimation in tea plants.

## 4. Materials and Methods

### 4.1. Plant Materials and Treatments

Potted tea plants of the cultivar ‘Longjing 43’ were grown at the Tea Research Institute, Chinese Academy of Agricultural Sciences, Hangzhou, Zhejiang Province, China. Three-year-old potted plants with uniform growth and no visible symptoms of pests or diseases were transferred to a controlled-environment growth chamber set at 25/20 °C (day/night), 70% relative humidity, a 14 h light/10 h dark photoperiod, and a light intensity of 300 μmol m^−2^ s^−1^. Plants were foliar-sprayed with a 100 nmol L^−1^ EBR solution, while control plants were sprayed with distilled water. Leaves were sprayed evenly on both sides until uniformly wetted without visible runoff, and the same spraying procedure was applied to all plants to ensure consistency among treatments. After three foliar applications at 12 h intervals, the plants were subjected to either normal temperature (25 °C) or low temperature (4 °C) treatment. Four treatment groups were established: control (Control), EBR treatment (EBR), low-temperature treatment (Cold), and combined EBR plus low-temperature treatment (Cold + EBR). For omics analyses, based on preliminary experiments, samples consisting of one bud with two leaves were collected from each plant at 12 and 48 h after the onset of temperature treatment. These two time points were selected to represent the relatively early and later stages of the response, respectively. Three biological replicates were included for each treatment.

### 4.2. RNA Isolation and Transcriptome Sequencing

Total RNA was extracted from leaf samples using the RNAprep Pure Plant Kit (DP441, Tiangen, Beijing, China). The purity, concentration, and integrity of the extracted RNA were assessed using a NanoPhotometer spectrophotometer (IMPLEN, Munich, Germany), a Qubit RNA Assay Kit (Life Technologies, Carlsbad, CA, USA), and an RNA Nano 6000 Assay Kit on the Bioanalyzer 2100 system (Agilent Technologies, Santa Clara, CA, USA), respectively. cDNA libraries were constructed and sequenced on an Illumina platform. Clean reads were mapped to the tea plant reference genome available in TPIA (https://tpia.teaplants.cn/, accessed on accessed on 20 September 2025). Gene expression abundance was expressed as FPKM values. DEGs were identified from read count data using DESeq2 v1.22.1, with thresholds of |log2(fold change)| ≥ 1 and adjusted *p*-value (*p*adj) < 0.05. Transcription factors were identified using iTAK (iTAK—Plant Transcription factor & Protein Kinase Identifier and Classifier, http://itak.feilab.net/cgi-bin/itak/index.cgi, accessed on 15 October 2025) based on the PlnTFDB and PlantTFDB databases.

### 4.3. Metabolomic Analysis

**Figure 7:**
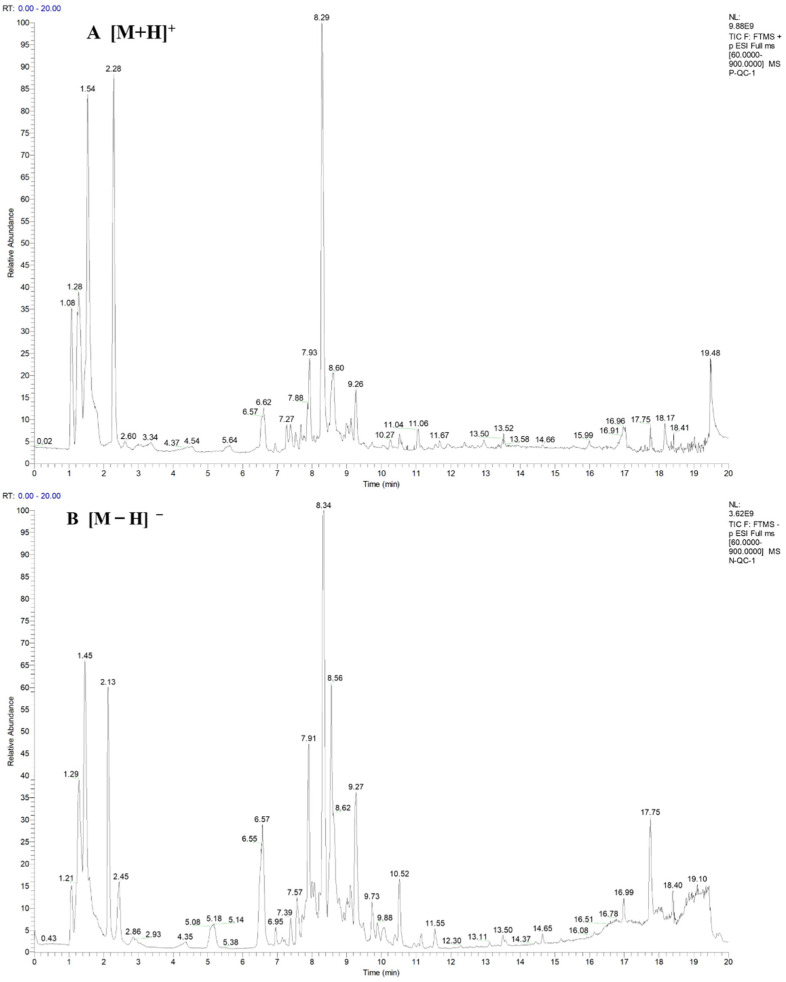
Full scan chromatograms of QC sample. (**A**) Positive ion mode of full scan chromatograms. (**B**) Negative ion mode of full scan chromatograms.

Tea leaves were lyophilized and ground into powder. A 0.1 g aliquot of the powder was extracted with 75% methanol and ultrasonicated at 15 °C. The supernatant was then filtered through a 0.22 μm membrane filter. Metabolites were analyzed using a UHPLC system (UltiMate 3000, Thermo Fisher Scientific, Waltham, MA, USA) equipped with a Waters ACQUITY HSS T3 column (Waters, Milford, MA, USA). The mobile phases consisted of 0.1% formic acid in water (solution A) and acetonitrile (solution B), and the elution program was performed as previously described [52]. Quality control (QC) samples were prepared by pooling equal aliquots of all samples. Mass spectrometric analysis was performed using a high-resolution quadrupole Orbitrap mass spectrometer (Q Exactive, Thermo Fisher Scientific, Waltham, MA, USA) equipped with an electrospray ionization (ESI) source operating in both positive and negative ion modes. The capillary voltage was set to 3500 V. Data were acquired in Full MS/dd-MS^2^ (TopN) mode over an *m*/*z* range of 60–900 (Figure 7). Raw data processing, including baseline correction, smoothing, noise reduction, and peak alignment, was performed using Xcalibur v2.1.1 and Compound Discoverer v3.2 (Thermo Fisher Scientific, Waltham, MA, USA). The data were filtered according to the following criteria: accurate mass deviation ≤ 5 ppm, retention time deviation ≤ 0.2 min, peak intensity ≥ 200,000, and signal-to-noise ratio > 3. To ensure data quality and stability, features with a relative standard deviation (RSD) of less than 25% in the QC samples were retained for further analysis. Metabolites were annotated using online databases, including the Human Metabolome Database (HMDB v5.0), mzCloud, and the Kyoto Encyclopedia of Genes and Genomes (KEGG) database. MetaboAnalyst v5.0 was used for metabolomics data analysis and visualization. DAMs were defined as those with a fold change ≥1.5 or ≤0.67 and adjusted *p*-value (*p*adj) < 0.05. Each treatment included three biological replicates.

### 4.4. Correlation Analysis Between DEGs and DAMs

An integrative correlation was performed using the Metware Cloud platform (https://cloud.metware.cn/, accessed on accessed on 28 November 2025) to examine their relationships analysis between DEGs and DAMs. Pearson correlation coefficients (PCCs) were calculated using normalized FPKM values of DEGs and the relative abundance of DAMs. Gene–metabolite pairs with |PCC| ≥ 0.9 and *p*adj < 0.05 were considered significantly correlated. Correlation networks were visualized using Cytoscape v3.9.1.

### 4.5. Total RNA Extraction, Quantitative Real-Time PCR (qRT-PCR) and Statistical Analysis

Total RNA was isolated from tea leaves using the RNAprep Pure Plant Plus Kit (TIANGEN, Beijing, China). Residual genomic DNA was removed according to the manufacturer’s instructions. First-strand cDNA was synthesized using the Evo M-MLV RT Kit (Accurate Biology, Changsha, Hunan, China). qRT-PCR was performed using the SYBR Green Premix Pro Taq HS qPCR Kit (Accurate Biology, Changsha, Hunan, China) on a LightCycler 480 II instrument (Roche, Basel, Switzerland). Relative gene expression levels were calculated using the 2^−ΔΔCt^ method [53] and normalized to *CsGAPDH* [54], which was used as the internal reference gene. Primer sequences used for qRT-PCR are listed in Table S2. All experiments were conducted with three independent biological replicates, and the data are presented as the mean ± standard deviation (SD). Statistical analyses were conducted using SPSS 26.0 (IBM Corp., Armonk, NY, USA). Significant differences among groups were determined by one-way analysis of variance (ANOVA) followed by Duncan’s multiple range test at *p* < 0.05.

## 5. Conclusions

This study provides a systems-level multi-omics view of cold acclimation in tea plants and suggests that brassinosteroid signaling acts primarily as a potentiator rather than an independent driver of this process. Integrated transcriptomic and metabolomic analyses showed that EBR application under cold conditions amplified endogenous cold-response programs, leading to coordinated reprogramming of a large number of genes and metabolites. At the metabolic level, phenylpropanoid biosynthesis emerged as a key pathway associated with protective responses, with EBR enhancing pathway-wide activation of upstream genes such as *PAL*, *C4H*, and *4CL*, together with the induction of lignin-associated genes (*CCR*, *CAD*) and flavonoid/catechin branch genes (*CHS*, *DFR*, *ANS*, *LAR*). This metabolic remodeling was accompanied by extensive hormone crosstalk, in which BR signaling interacted with jasmonic acid- and abscisic acid-related pathways to modulate growth–stress balance under low temperature. In addition, TF–metabolite-gene correlation analysis identified candidate hub genes, including WRKY transcription factor genes (TEA001162, TEA027058) and a UDP-glycosyltransferase gene (TEA025792), that were closely associated with phenylpropanoid-centered metabolic modules. Together, these findings provide a useful resource for understanding coordinated transcript–metabolite regulation during cold acclimation and offer a theoretical basis for breeding and cultivation strategies aimed at improving cold tolerance in tea plants.

## Data Availability

All data are available within the manuscript and its supporting materials. The raw transcriptome sequencing data generated in this study have been deposited in the NCBI Sequence Read Archive (SRA) under BioProject accession number PRJNA1451402.

## Supplementary Materials

The following supporting information can be downloaded at: https://www.mdpi.com/article/10.3390/ijms27093766/s1.
