# Supplementary material for: Brassinosteroid Potentiates Cold-Induced Transcriptome–Metabolome Reprogramming in Tea Plant Leaves: An Integrated Multi-Omics Landscape
Source: Int J Mol Sci. 2026 Apr 23;27(9):3766. doi: 10.3390/ijms27093766 (PMC13164448; doi:10.3390/ijms27093766)
Supplement: Supplementary file 1 [file ijms-27-03766-s001.zip › ijms-4268553-supplementary.pdf]

Table S1. Quality of transcriptome sequencing.

All RNA-seq libraries showed high sequencing quality, with Q30 values above 96% and GC contents around 45%, indicating that the sequencing data were of sufficient quality for subsequent analyses.

Sample	Raw Reads (M)	Raw Bases (Gb)	Clean Reads (M)	Clean Bases (Gb)	Valid Bases (%)	Q30 (%)	GC (%)
Control-1	41.63	6.05	40.3	5.86	96.8	96.88	45.24
Control-2	46.21	6.69	44.54	6.45	96.38	97.29	45.23
Control-3	42.53	6.18	41.11	5.97	96.66	96.82	45.25
EBR-1	49.8	7.19	47.85	6.91	96.08	97.05	45.25
EBR-2	47.58	6.89	45.88	6.65	96.42	97.09	45.2
EBR-3	46.87	6.79	45.17	6.54	96.37	96.74	45.31
Cold-1	50.51	7.28	48.41	6.98	95.84	97.21	45.22
Cold-2	47.96	6.95	46.23	6.7	96.39	96.58	45.45
Cold-3	49.86	7.22	48.02	6.95	96.31	96.28	45.36
Cold+EBR-1	49.68	7.2	47.88	6.94	96.39	96.79	45.28
Cold+EBR-2	51.12	7.34	48.81	7.01	95.49	96.68	45.37
Cold+EBR-3	50.39	7.28	48.44	7	96.14	96.86	45.37

Table S2. Primers for RT-qPCR assays.

Gene/ID	Forward sequence	Reverse sequence (5'-3')
CsGAPDH	TTGGCATCGTTGAGGGTCT	CAGTGGGAACACGGAAAGC
TEA028429	GGCGGTTGGTACTCTTTCCA	GCGACGCTATCAGATGTGGA
TEA025792	GTTCTGGGTCTGGAGCTCAC	CACAGAAGGGTGCGCTAGAA
TEA001162	GTTCAAGCGACGTCGAATCG	GCAAAACGTGGCTGTTGGAT
TEA027058	ATGTCAAGCTGAGGTGGCTC	GGTTGAAATGGCTGCTGCAA
TEA003137	TGTCATGGCCAGCAGTGATT	CCTTGTAGCACAGTGAGGCA
TEA024587	AGTCGCCAAACGCACTCTAA	CGTGCACTCTCCACCTCTTT
TEA034012	AGGCAGTTGCAGCTGATGAT	GTGCTTGCTTGGTTCCTGTG
TEA023333	GCTAGGCAGGACATGGTTGT	TTGGCTAAGCGAAGAACCGT
TEA034003	TTCATTTCCCCGCCTCTTCC	CAGTTGAAGGAGACACCGCT
TEA019177	CCACAAACCAAGCTTCCACG	TGAGCTGCCTCAAATGCTGA
TEA001818	GCATTTGCTCGGTGTTTGGT	ATTTCCTCGCCATCGGTGTT
TEA017407	CTGGTTCACTCACATGGCCT	CAACGAGCACAACCCCAAAG

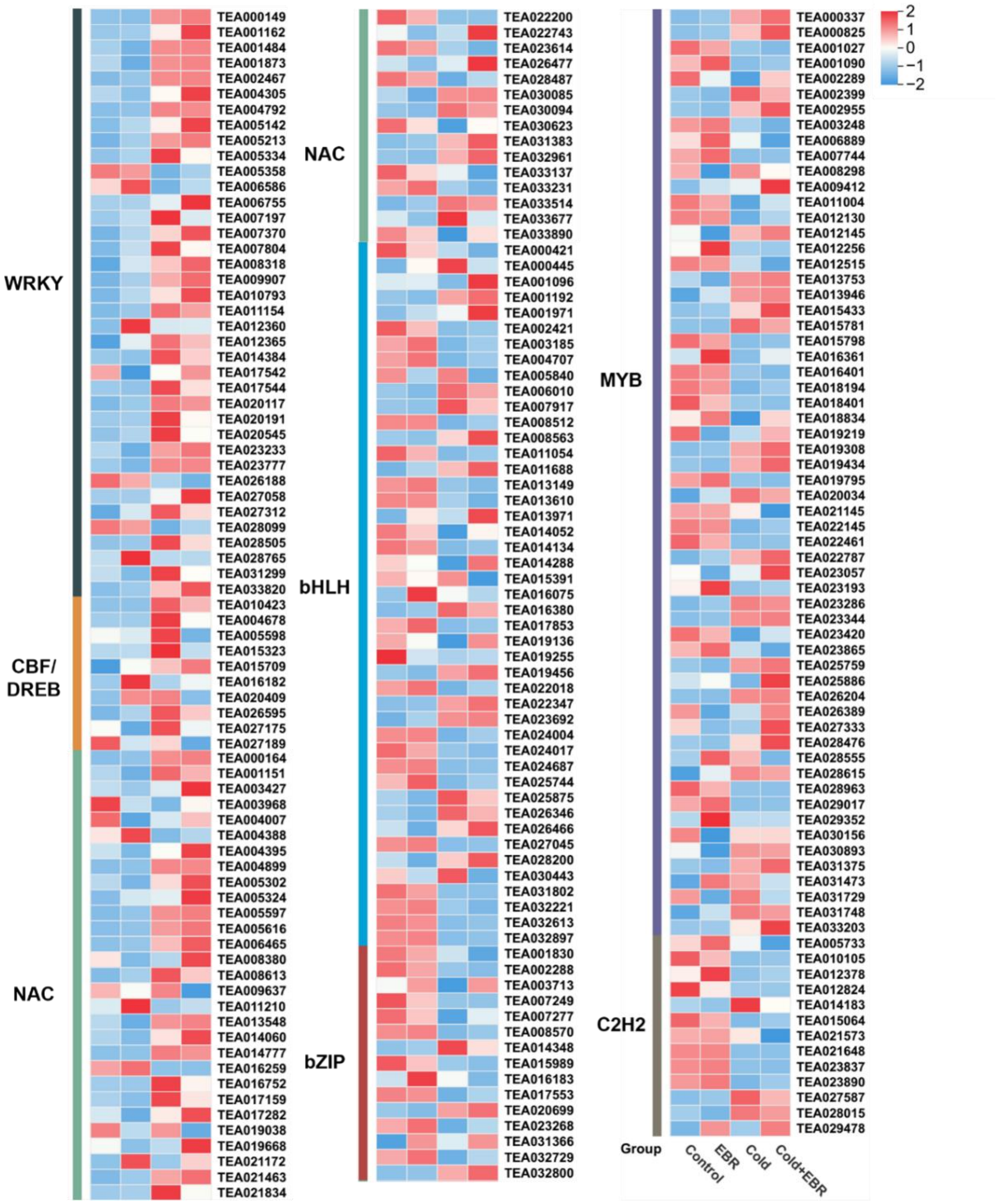

Figure S1. Heatmap of differentially expressed genes (DEGs) encoding transcription factors under different treatments. DEGs belonging to different transcription factor families, including WRKY, NAC, bHLH, CBF/DREB, bZIP, MYB, and C2H2, are shown. Columns represent the Control, EBR, Cold, and Cold + EBR treatments. Heatmap values were generated from FPKM data and further normalized in a row-wise manner. Red and blue indicate relatively high and low expression levels, respectively.

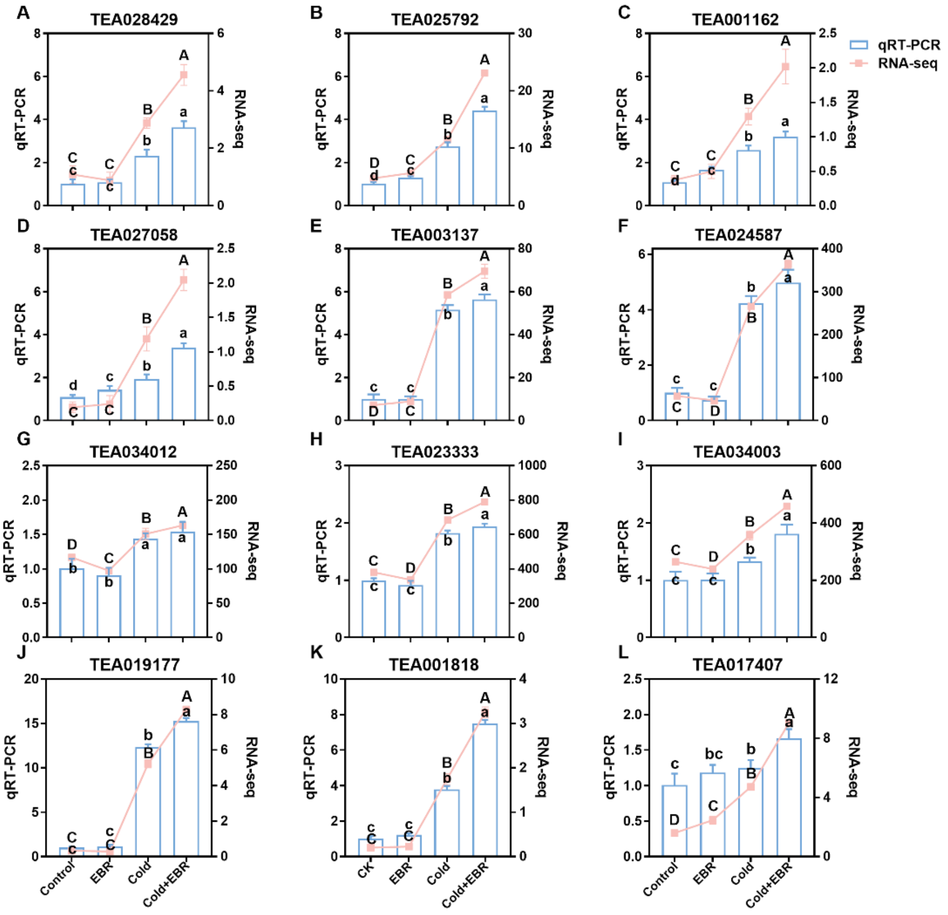

Figure S2. qRT-PCR validation of RNA-seq results. Expression profiles of 12 selected genes under Control, EBR, Cold, and Cold+EBR treatments: (A) TEA028429, (B) TEA025792, (C) TEA001162, (D) TEA027058, (E) TEA003137, (F) TEA024587, (G) TEA034012, (H) TEA023333, (I) TEA034003, (J) TEA019177, (K) TEA001818, and (L) TEA017407. qRT-PCR data are shown as blue bars (left y-axis), and RNA-seq data are shown as a pink line (FPKM, right y-axis). Values are presented as mean ± SD (n = 3). Different lowercase and uppercase letters indicate significant differences among treatments in qRT-PCR and RNA-seq data, respectively (one-way ANOVA followed by LSD test, $P < 0.05$).
